# Morbidity and mortality in primary versus secondary antiphospholipid syndrome: A single-center study using the 2023 ACR/EULAR criteria

**DOI:** 10.1177/09612033261449991

**Published:** 2026-05-06

**Authors:** Roni Meidan, Monica Angerri Nadal, Blanca Viejo Sosa, Uxia Couto Lareo, Prabal Mittal, Hannah Cohen, David Isenberg

**Affiliations:** 1 Department of Rheumatology, 26738 Tel Aviv Sourasky Medical Center, Tel Aviv, Israel; 2 Gray Faculty of Medical and Health Sciences, Tel Aviv University, Tel Aviv, Israel; 3 Servei Medicina Interna, Hospital Universitario Dr Joseph Trueta, Girona, Spain; 4 Servicio de Reumatologia, Hospital Universitario Jerez de la Frontera, Cadiz, Spain; 5 Servizo de Reumatoloxia, 16811 Complexo Hospitalario Universitario A Coruna, A Coruna, Spain; 6 Department of Hematology, The Royal Free Hospital NHS Foundation Trust, London, UK; 7 Department of Hematology, University College London Hospitals NHS Foundation Trust, London, UK; 8 Department of Ageing, Rheumatology and Regenerative Medicine, Division of Medicine, University College London, London, UK

**Keywords:** systemic lupus erythematosus, antiphospholipid syndrome, morbidity, mortality

## Abstract

**Objective:**

To evaluate differences in morbidity and mortality between patients with primary antiphospholipid syndrome (PAPS) and APS secondary to systemic lupus erythematosus (SAPS), classified according to the 2023 ACR/EULAR APS criteria.

**Methods:**

A single-center retrospective observational study including consecutive adult patients evaluated for suspected APS, retrospectively classified according to the 2023 ACR/EULAR APS criteria. Those patients who were included were categorized as SAPS based on the presence of concomitant SLE, defined using the 2012 Systemic Lupus International Collaborating Clinics criteria. Demographic, clinical, laboratory, and outcome data were extracted from medical records. Group comparisons were performed using t-tests, chi-square or Fisher’s exact tests. Multivariable Cox regression was used to identify factors associated with mortality.

**Results:**

Of 432 patients who were screened, 210 (48.6%) fulfilled the 2023 ACR/EULAR criteria and were included, comprising 151 (71.9%) with PAPS and 59 (28.1%) with SAPS. Compared with PAPS, SAPS patients were more frequently female (91.7% vs 65.6%, p < 0.001), of younger age at first APS-related event (34.2 vs 43.1 years, p < 0.001), and had a higher prevalence of renal disease (33. 9% vs 6.0%, p < 0.001). Rates of venous, arterial, recurrent thrombosis, and obstetric manifestations were similar between groups. CAPS and overall mortality were higher in SAPS (11.9% vs 2.7%, p = 0.01; 22.0% vs 10.6%, p = 0.045, respectively). In multivariable Cox regression, increasing age at first event, SAPS, and renal disease were independently associated with increased mortality (adjusted HR 1.1, 5.28, and 3.16, respectively).

**Conclusion:**

SAPS is associated with higher mortality and increased CAPS risk, emphasizing the adverse prognostic impact of concomitant SLE and the need for tailored risk stratification.

## Introduction

Antiphospholipid syndrome (APS) is a systemic autoimmune disorder characterized by thrombotic and obstetric manifestations occurring in the presence of persistent antiphospholipid antibodies (aPL), including lupus anticoagulant (LA), anticardiolipin (aCL), and anti-β2 glycoprotein I (aβ2GLI) antibodies.^1^ APS is associated with substantial long-term morbidity, including venous thromboembolic events, most commonly deep vein thrombosis (DVT) and pulmonary embolism (PE); arterial thrombosis, particularly ischemic stroke and obstetric morbidity, such as fetal death, pre-eclampsia and placental insufficiency.^2^

APS was previously classified according to the revised Sapporo criteria, combining clinical manifestations with laboratory evidence of persistent aPL.^3^ In 2023, the American College of Rheumatology (ACR) and the European Alliance of Associations for Rheumatology (EULAR) introduced new classification criteria to improve diagnostic accuracy and standardization. These updated criteria incorporate weighted clinical and laboratory domains, providing greater specificity and an improved framework for future research.^4^ Notably, much of the existing literature examining morbidity and mortality in APS predates the implementation of these updated criteria.

APS may occur as a primary condition (primary APS, PAPS) or in association with other autoimmune diseases, most commonly systemic lupus erythematosus (secondary APS, SAPS).^5^ Although concomitant SLE does not appear to influence long-term thrombotic recurrence or aPL profiles significantly,^6,7^ several studies have reported higher overall morbidity and all-cause mortality in SAPS compared with PAPS.^8,9^ However, much of the available evidence derives from retrospective cohorts and narrative reviews conducted under earlier classification frameworks, and contemporary data evaluating long-term outcomes in PAPS and SAPS remain limited.^10,11^

Accordingly, this study aimed to compare the clinical characteristics of patients with PAPS versus those with SAPS utilizing current criteria, with a particular focus on differences in overall morbidity and all-cause mortality. In addition, we sought to identify potential predictors of adverse clinical outcomes, including aPL serological profiles and relevant comorbidities.

## Methods

This single-center, retrospective observational study included consecutive patients managed in the Rheumatology and Hematology clinics at university college hospital over a period of approximately four decades (1978–2025). Patients were identified through established departmental clinical databases, and demographic, clinical, and laboratory data were collected through detailed review of paper and electronic medical records. Follow-up time was calculated from the first APS-related clinical event to death or last documented clinical contact.

Individuals aged 18 years or older who fulfilled the 2023 ACR/EULAR classification criteria for APS were eligible for inclusion. The criteria were applied retrospectively by the treating hematology physicians where available, and otherwise by the study authors following standardized review of the medical records. Patients with insufficient data to apply the classification criteria reliably or with borderline findings were excluded. Concomitant SLE was defined according to the 2012 Systemic Lupus International Collaborating Clinics (SLICC) classification criteria, as the retrospective nature of the cohort and incomplete data limited consistent application of the 2019 EULAR/ACR criteria. Patients with obstetric-only APS were also excluded from the final analysis due to their relatively small sample size, which precluded meaningful outcome analysis.

Thromboembolic events were classified as venous or arterial and were confirmed primarily by imaging studies, including duplex ultrasonography, computed tomography, or magnetic resonance imaging, as appropriate. Venous thromboembolic events included DVT, PE, cerebral venous sinus thrombosis (CVST), and other venous thromboses, while arterial events included ischemic stroke, myocardial infarction, and peripheral arterial thrombosis. Recurrent thrombosis was defined as the occurrence of more than one thromboembolic event during follow-up. Catastrophic antiphospholipid syndrome (CAPS) was diagnosed in accordance with established classification criteria.^12^

Renal involvement was analyzed as a composite outcome; where available, underlying diagnoses included lupus nephritis, thrombotic microangiopathy, or other renal impairment.

Laboratory variables were assessed using standardised laboratory testing, including complete blood count and aPL testing (lupus anticoagulant (LA), aCL IgG/IgM, and aβ2GPI IgG/IgM).^13^ aCL and B2GP1 IgG and IgM antibodies were assessed by solid phase immunoassays (ELISA- or chemiluminescence-based), and LA by clot-based assays, in accordance with (contemporaneous) international recommendations.^14^ Antibody titers ≥40 units were considered positive.^4^ Persistent aPL positivity was defined as the presence of the same antibody on two or more occasions at least 12 weeks apart.^15^

### Ethics statement

This study used fully anonymized data collected as part of routine clinical care and was classified as a clinical audit/service evaluation, for which formal research ethics committee approval was not required.

### Statistical analyses

Continuous variables were presented as means ± standard deviations and compared between groups using the t-test. Categorical variables were expressed as frequencies and percentages and compared using Fisher’s exact test or chi-square test.

All statistical tests were two-sided, and a p-value of less than 0.05 was considered statistically significant.

Time-to-event analysis was performed to assess all-cause mortality, with follow-up defined from the date of the first documented APS-related clinical event until death or the end of the study period. Deaths were ascertained through review of hospital medical records and clinical documentation. Survival curves were generated using the Kaplan–Meier method and compared between groups using the log-rank test.

A multivariable Cox regression analysis was performed to identify factors independently associated with all-cause mortality. Candidate variables were selected a priori based on clinical relevance and data availability. Univariate analyses were used to explore associations with mortality, and clinically relevant variables were entered into the multivariable model together with key demographic covariates. For time-to-event analyses, one patient who died during follow-up was excluded from the Cox regression model due to missing data about time of the first APS-related event.

Exploratory time-to-event analyses were performed for recurrent thrombosis, CAPS, and 10-years mortality; however, due to the limited number of events, these analyses were underpowered and were not included in the primary analyses.

## Results

### Baseline characteristics

**Figure 1.**
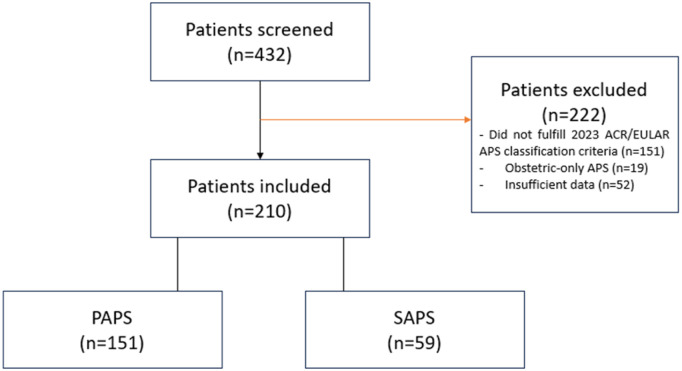
Flow diagram of the study cohort. Consecutive adult patients with suspected APS were retrospectively reclassified according to the 2023 ACR/EULAR criteria; patients not meeting criteria, with insufficient data, or with obstetric-only manifestations were excluded. The final cohort was stratified into primary APS (PAPS) and secondary to SLE APS (SAPS).

A total of 432 patients with suspected APS were screened, of whom 210 (48.6%) met the inclusion criteria and were included in the final analysis. Patients were excluded due to insufficient data (n = 52), obstetric-only APS (n = 19), or failure to fulfill strictly the 2023 ACR/EULAR APS classification criteria (n = 151). Failure to meet these criteria was most commonly due to not fulfilling the required clinical and/or laboratory domain thresholds (Figure 1). Of the patients included, 151 (71.9%) had PAPS and 59 (28.1%) had SAPS. Patients with SAPS were more frequently female than those with PAPS (91.7% vs 65.6%, p < 0.001). Mean body mass index did not differ significantly between groups. Ethnic distribution differed between groups (p = 0.02), with a higher proportion of white patients in the PAPS group. Among patients with renal involvement (n = 29), lupus nephritis was the most common diagnosis (n = 14), followed by thrombotic microangiopathy (n = 6), with one patient demonstrating overlapping features; the remaining cases comprised renal involvement without a specific histopathological classification, including chronic kidney disease likely attributable to alternative etiologies (e.g., diabetes mellitus, hypertension, or polycystic kidney disease). Overall, renal involvement was more common in SAPS than in PAPS (33.9% vs 6.0%, p < 0.001). Smoking status and cardiovascular comorbidities were comparable between groups. Baseline characteristics are summarized in Table 1.

**Table 1.** Baseline characteristics of patients with primary and secondary APS.

Variable	PAPS (n = 151)	SAPS (n = 59)	P-value
Female sex, n (%)	99 (65.6)	55 (91.7)	**<0.001**
BMI, kg/m^2^, mean ± SD	28.7 ± 6.6	27.99 ± 6.3	0.38
Ethnicity, n (%)			**0.02**
White	104 (68.9)	27 (45.0)	
Asian	14 (9.3)	10 (16.7)	
Black	9 (6.0)	8 (13.3)	
Other or not available data	24 (15.8)	14 (23.7)	
Smoker, n (%)	28 (18.5)	12 (20.3)	0.80
Hypertension, n (%)	32 (21.2)	12 (20.3)	0.93
Dyslipidemia, n (%)	29 (19.2)	12 (20.3)	0.89
Diabetes mellitus, n (%)	10 (6.6)	5 (8.5)	0.39
Atrial fibrillation, n (%)	10 (6.6)	3 (5.1)	0.76
Kidney disease, n (%)	9 (6.0)	20 (33.9)	**<0.001**
Malignancy, n (%)	13 (8.6)	3 (5.1)	0.57
Immune thrombocytopenia, n (%)	3 (2.0)	5 (8.5)	**0.04**

PAPS, primary antiphospholipid syndrome; SAPS, SLE related antiphospholipid syndrome; BMI, body mass index.

### Clinical manifestations

Clinical manifestations are presented according to the major domains of the 2023 ACR/EULAR APS classification criteria, including macrovascular thrombosis (venous and arterial), microvascular involvement, obstetric manifestations, cardiac involvement, hematologic features, and laboratory findings. Clinical and laboratory characteristics are summarized in Table 2.

**Table 2.** Clinical and laboratory characteristics of patients with primary and secondary APS.

Variable	PAPS (n = 151)	SAPS (n = 59)	P-value
Clinical manifestations			
Age at first event (years), mean ± SD	43.1 ± 15.6	34. 2 ± 15.1	**<0.001**
Macrovascular (venous), n (%)	88 (58.3)	33 (55.9)	0.664
Deep vein thrombosis, n (%)	50 (33.1)	24 (40.7)	0.344
Pulmonary embolism, n (%)	50 (33.1)	15 (25.4)	0.25
Cerebral venous sinus thrombosis, n (%)	13 (8.6)	3 (5.1)	0.56
Other venous thrombosis, n (%)	10 (6.6)	7 (11.9)	0.17
Macrovascular (arterial), n (%)	82 (54.3)	28 (47.4)	0.43
Stroke, n (%)	73 (48.3)	27 (45.8)	0.82
Myocardial infarction, n (%)	3 (2.0)	3 (5.1)	0.52
Peripheral arterial thrombosis, n (%)	11 (7.3)	3 (5.1)	0.45
Microvascular, n (%)	8 (5.3)	8 (13.5)	0.13
Livedo racemosa, n (%)	0.0	0.0	1.00
Digital ischemia, n (%)	3 (2.0)	4 (6.8)	1.00
Adrenal hemorrhage, n (%)	2 (1.3)	0 (0)	1.00
Pulmonary hemorrhage, n (%)	0 (0)	1 (1.7)	0.63
Nephropathy, n (%)	3 (1.98)	3 (5.08)	0.35
Obstetric, n (%)	25 (16.5)	6 (10.2)	0.31
Cardiac, n (%)	3 (2.0)	2 (3.4)	0.62
Hematology, n (%)	10 (6.6)	7 (11.9)	0.26
Laboratory			
LA, n (%)	124 (82.1)	48 (81.3)	0.93
Mod-high titer IgM β2GP1, n (%)	26 (17.2)	12 (20.3)	0.63
Mod-high titer IgG β2GP1, n (%)	74 (49.0)	34 (57.6)	0.25
Mod-high titer IgM aCL, n (%)	23 (15.2)	14 (23.7)	0.16
Mod-high titer IgG aCL, n (%)	75 (49.7)	29 (49.1)	0.69
Triple positive, n (%)	59 (39.1)	26 (44.1)	0.72

PAPS, primary antiphospholipid syndrome; SAPS, SLE related antiphospholipid syndrome; LA, lupus anticoagulant; β2GP1, β2 glycoprotein 1; aCL, anti cardiolipin.

#### Macrovascular thrombosis

Patients in the SAPS group experienced their first APS-related thrombotic event (venous or arterial) at a younger age compared with those in the PAPS group (34.2 ± 15.1 vs 43.1 ± 15.6 years; p < 0.001).

#### Macrovascular (venous thromboembolism)

Macrovascular venous thrombosis was common and occurred at similar rates in patients with PAPS and SAPS (58.3% vs 55.9%, p = 0.664). DVT was the most frequent venous manifestation, affecting 33.1% of patients with PAPS and 40.7% of those with SAPS (p = 0.344), followed by PE (33.1% vs 25.4%, p = 0.25). CVST was observed in 8.6% of PAPS patients and 5.1% of SAPS patients (p = 0.56). Other venous thrombotic events were uncommon and did not differ significantly between groups.

#### Macrovascular (arterial thrombosis)

Macrovascular arterial events occurred in a substantial proportion of patients in both groups, with no significant difference between PAPS and SAPS (54.3% vs 47.45%, p = 0.43). Ischemic stroke was the predominant arterial manifestation and occurred at comparable frequencies in the two groups (48.3% vs 45.8%, p = 0.82). Myocardial infarction and peripheral arterial thrombosis were uncommon and did not differ significantly between groups.

#### Microvascular

Microvascular involvement was infrequent overall but numerically more common in patients with SAPS than in those with PAPS (13.5% vs 5.3%, p = 0.13). Livedo racemosa was not observed in either group. Adrenal hemorrhage was observed only in two patients with PAPS, while suspected pulmonary hemorrhage occurred in a single patient with SAPS. APS nephropathy was rare and similarly distributed between groups (1.98% vs 5.08%, p = 0.35).

#### Obstetric

Obstetric manifestations occurred in 16.6% of patients with PAPS and 10.2% of those with SAPS, with no significant difference between groups (p = 0.31).

#### Cardiac involvement

Cardiac involvement, including valvular abnormalities, was uncommon and was observed in 2.0% of patients with PAPS and 3.4% of those with SAPS, with no significant difference between groups (p = 0.62).

### Hematological manifestations

Thrombocytopenia, the only hematological manifestation, was observed in 6.6% of patients with PAPS and 11.9% of those with SAPS, with no significant difference between groups (p = 0.26).

### Laboratory findings

The prevalence of lupus anticoagulant (LA), anticardiolipin (aCL IgG and IgM), and anti-β2 glycoprotein I (aβ2GPI IgG and IgM) antibodies did not differ significantly between PAPS and SAPS. Likewise, the prevalence of moderate-to high-titer antibodies (≥40 units) and the rate of triple-positive serology did not differ significantly between groups.

### Clinical outcomes

Clinical outcomes are summarized in Table 3

**Table 3.** Thrombotic recurrence, catastrophic APS, and mortality in primary and secondary APS.

Variable	PAPS (n = 151)	SAPS (n = 59)	P-value
Recurrent thrombi, n (%)	66 (44.4)	28 (47.5)	0.60
Catastrophic APS, n (%)	4 (2.6)	7 (11.9)	**0.01**
Death, n (%)	16 (10.6)	13 (22.0)	**0.045**
Mean age at death (years)	66.9 ± 16.0	61.8 ± 9.4	0.32
Death within 10 years from first event, n (%)	6 (4.0)	6 (10.0)	0.10

APS, antiphospholipid syndrome; PAPS, Primary antiphospholipid syndrome; SAPS, SLE related antiphospholipid syndrome.

#### Recurrent thrombosis

Recurrent thrombotic events occurred in 94 patients (45.8%) across the cohort, including 66 (44.4%) in the PAPS group and 28 (47.5%) in the SAPS group, and did not differ significantly between groups (p = 0.60). In the PAPS cohort, recurrent thrombosis was more frequent among triple-positive patients compared with non–triple-positive patients (54.2% vs 36.9%, p = 0.04), whereas no such association was observed in the SAPS cohort (46.2% vs 48.5%, p = 0.86).

#### Catastrophic APS

CAPS occurred in 11 patients (5.2%), including 4 patients (2.6%) in the PAPS group and 7 patients (11.9%) in the SAPS group. CAPS was significantly more frequent in the SAPS group (p = 0.01).

#### Mortality

Baseline characteristics stratified by mortality status are summarized in Supplement Table S1.

Overall, 29 patients (13.8%) died during follow-up; One death was excluded from time-to-event analyses due to missing information about the time of the first APS-related event. Mortality was higher in the SAPS group compared with the PAPS group (22.0% vs 10.6%), and this difference was statistically significant (p = 0.045). The mean age at death did not differ significantly between groups, with patients in the PAPS group dying at a mean age of 66.9 ± 16.0 years compared with 61.8 ± 9.4 years in the SAPS group (p = 0.32). Recorded causes of death included infection, stroke, intracranial hemorrhage, malignancy, and cardiac complications.

**Figure 2.**
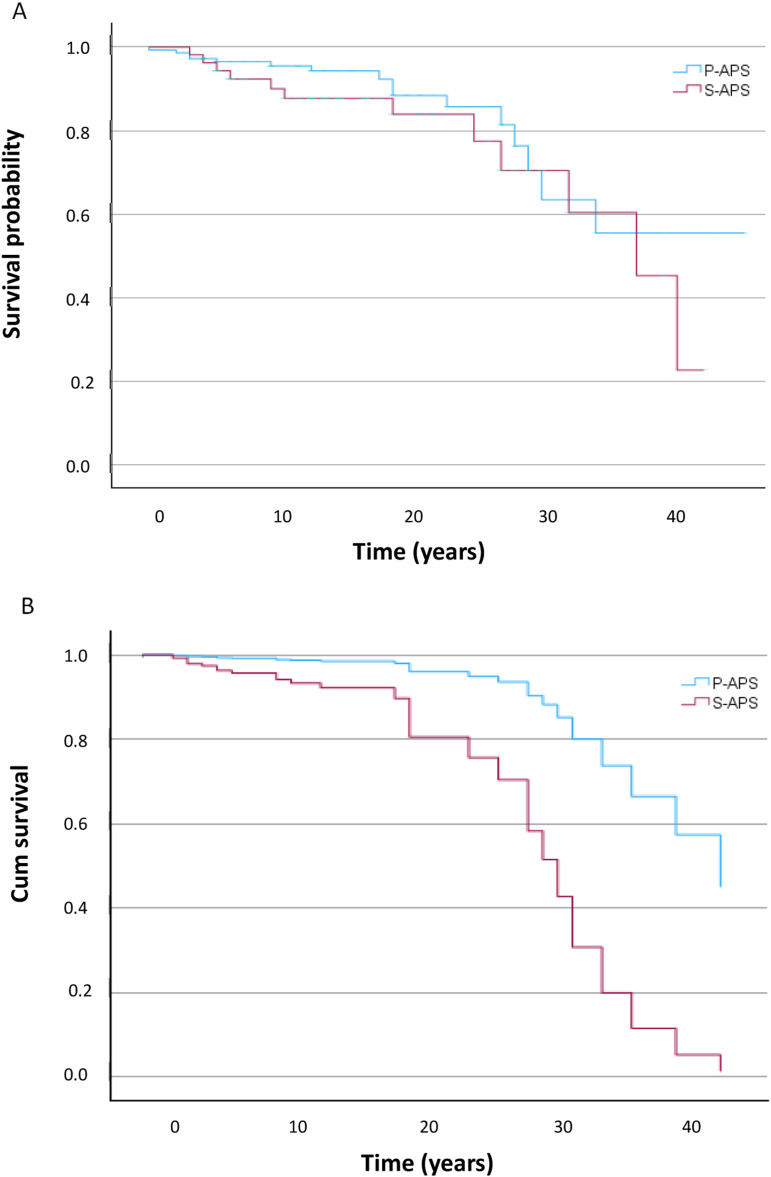
Overall survival in primary and secondary APS over time (reference time from first event) (A) Kaplan–Meier curves stratified by APS subtype (log-rank p = 0.28). (B) Cox model–adjusted survival curves showing predicted survival by APS subtype adjusted for age at first event and covariates included in the multivariable model.

Kaplan–Meier survival analysis stratified by APS subtype showed overlapping survival curves for patients with primary and secondary APS, with no statistically significant difference in overall survival on log-rank testing (p = 0.28) (Figure 2).

Given differences in baseline characteristics between groups, adjusted analyses were performed. In multivariable Cox regression analysis, increasing age at first APS-related event was associated with higher mortality (adjusted HR 1.08 per year, 95% CI 1.04–1.12; p < 0.001). SAPS was associated with a significantly increased risk of death compared with PAPS (adjusted HR 5.28, 95% CI 1.80–15.52; p = 0.002). Kidney disease was also independently associated with increased mortality (adjusted HR 3.16, 95% CI 1.11–8.95; p = 0.031). These results should be interpreted in the context of a limited number of events. Full model estimates are presented in Table 4 and Figure 2.

**Table 4.** Multivariable Cox regression analysis for mortality.

Variable	Adjusted HR	95% CI	P-value
Gender (male vs female)	2.1	0.67–6.4	0.21
White ethnicity	2.5	0.79–7.9	0.12
Atrial fibrillation	3.5	1.05–11.4	**0.04**
Kidney disease	3.2	1.1–8.9	**0.031**
Malignancy	1.2	0.3–4.1	0.76
SAPS vs PAPS	5.3	1.79–15.5	**0.002**
Age at first APS-related event (per-year)	1.1	1.04–1.12	**<0.001**
Digital ischemia	6.0	1.41–25.2	**0.015**

HR, hazard ratio; CI, confidence interval; PAPS, Primary antiphospholipid syndrome; SAPS, SLE related antiphospholipid syndrome.

Total events = 28.

## Discussion

This retrospective study compared the clinical, laboratory, and outcome characteristics of patients with primary and secondary APS over four decades of follow-up. We found that patients with SAPS were more frequently female and exhibited a distinct clinical phenotype, characterised by a higher prevalence of kidney disease, an earlier age at first APS-related thrombotic event, and a higher incidence of CAPS and mortality. These findings highlight important differences in disease burden and prognosis between APS subtypes.

Baseline differences between the two APS subtypes were consistent with established epidemiological and clinical features of SLE. Patients with SAPS were more frequently female, reflecting the well-recognised female predominance of SLE, and APS-related clinical events occurred at a younger age than patients with primary APS. Renal involvement was substantially more common in SAPS, including lupus nephritis and advanced chronic kidney disease, most likely reflecting the contribution of underlying SLE rather than the APS alone.^16,17^ Previous SLE cohorts have demonstrated that the coexistence of APS is associated with increased irreversible organ damage and greater non-thrombotic morbidity, and reduced survival, further supporting the clinical relevance of distinguishing these phenotypes.^18,19^

Despite baseline differences, the clinical phenotype of APS was broadly similar in both primary and secondary disease. Arterial and venous thrombosis, including recurrence, as well as obstetric morbidity, occurred at comparable rates, and aPL profiles were largely overlapping. These observations align with APS ACTION registry data demonstrating that while SLE modifies certain clinical features, it does not substantially alter the core thrombotic manifestations among aPL-positive patients.^7^

Accordingly, the observed differences in clinical outcomes between primary and secondary APS are unlikely to be explained by thrombotic burden alone, as rates of recurrent thrombosis were comparable between groups. Instead, the higher incidence of CAPS and mortality in SAPS may reflect the cumulative effects of systemic inflammation, immune dysregulation, and end-organ involvement associated with underlying SLE, together with greater susceptibility to infection and treatment-related complications.^18,20^

Consistent with these observations, in this cohort, patients with SAPS experienced significantly higher mortality than those with PAPS. Although Kaplan–Meier analyses did not demonstrate a statistically significant difference in unadjusted survival, multivariable Cox regression identified SAPS as an independent predictor of mortality after accounting for differences in baseline characteristics. The apparent late divergence of adjusted survival curves likely reflects model-based predictions in the context of limited numbers at risk at long follow-up, rather than observed survival differences.

This study has several limitations. Its retrospective design introduces the potential for selection bias, and incomplete availability of clinical and laboratory data for some patients may have affected the completeness of phenotypic characterisation. Although the cohort was relatively large, several clinical manifestations—particularly microvascular, cardiac, and obstetric features—were infrequent, limiting statistical power for subgroup analyses and multivariable modeling. Inclusion was restricted to patients fulfilling the 2023 ACR/EULAR classification criteria for APS; therefore, individuals with isolated non-thrombotic or non-obstetric features – such as microvascular or hematological manifestations – who did not meet full classification thresholds were not included and may be underrepresented. In addition, a small proportion of patients had APS in the context of other autoimmune rheumatological conditions, introducing clinical heterogeneity. Given the limited numbers and heterogeneity within this subgroup, separate analyses were not performed.

In contrast, this study has several strengths, including a relatively large cohort with exceptionally long-term follow-up, with patients assessed consistently over several decades by the senior authors (HC and DI). The application of the 2023 ACR/EULAR classification criteria provides a contemporary and standardized framework, enhancing diagnostic specificity and comparability with future studies. The single-center design enabled comprehensive and internally consistent evaluation of clinical and laboratory manifestations, allowing a detailed comparison between primary and secondary APS.

## Conclusion

In this large, long-term single-center cohort classified according to the 2023 ACR/EULAR criteria, APS secondary to SLE was associated with a distinct clinical phenotype and worse outcomes compared with primary APS. Patients with SAPS had earlier disease onset, a higher burden of renal involvement, and a higher incidence of catastrophic APS and mortality, despite comparable thrombotic recurrence and similar antiphospholipid antibody profiles. These findings highlight the prognostic importance of concomitant SLE in APS and suggest that SAPS represents a higher-risk population that may benefit from closer surveillance and individualized management. Further studies are needed to refine risk stratification and optimize management strategies in this subgroup.

## Supplemental material

Supplemental material - Morbidity and mortality in primary versus secondary antiphospholipid syndrome: A single-center study using the 2023 ACR/EULAR criteria Supplemental Material for Morbidity and mortality in primary versus secondary antiphospholipid syndrome: A single-center study using the 2023 ACR/EULAR criteria by Roni Meidan, Monica Angerri Nadal, Blanca Viejo Sosa, Uxia Couto Lareo, Prabal Mittal, Hannah Cohen, David Isenberg in Lupus.

## Data Availability

The datasets generated and/or analyzed during the current study are not publicly available due to institutional data protection regulations but are available from the corresponding author on reasonable request and subject to appropriate ethical and data governance approvals.*
