# Supplementary material for: Morbidity and mortality in primary versus secondary antiphospholipid syndrome: A single-center study using the 2023 ACR/EULAR criteria
Source: Lupus. 2026 May 6;35(9):918–26. doi: 10.1177/09612033261449991 (PMC13320137; doi:10.1177/09612033261449991)
Supplement: Supplemental material - Morbidity and mortality in primary versus secondary antiphospholipid syndrome: A single-center study using the 2023 ACR/EULAR criteria [file sj-pdf-1-lup-10.1177_09612033261449991.pdf]

Table S1. Baseline characteristics stratified by mortality status

**Variable**	**Alive at last follow-up (n=181)**	**Deceased during follow-up (n=29)**	**P-value**
Female sex, n (%)	132 (72.9)	22 (75.9)	0.74
Ethnicity white	74 (40.9)	5 (17.2)	**0.01**
Body mass index, kg/m²	28.7 ± 6.5	26.2 ± 6.2	0.13
Hypertension, n (%)	33 (18.3)	11 (37.9)	**0.02**
Dyslipidemia, n (%)	32 (17.7)	8 (27.6)	0.21
Diabetes Mellitus, n (%)	11 (6.1)	4 (13.8)	0.13
Atrial Fibrillation, n (%)	6 (3.3)	7 (24.1)	**<0.001**
Kidney disease, n (%)	20 (11.0)	9 (31.0)	**0.004**
Malignancy, n (%)	11 (6.1)	5 (17.2)	**0.035**
Age at first event (years)	39.4 ± 15.8	48.4 ± 17.21	**0.005**
Macrovascular (venous), n (%)	102 (56.4)	19 (65.5)	0.35
Macrovascular (arterial), n (%)	94 (51.9)	16 (55.2)	0.74
Recurrent thrombi	77 (42.5)	17 (60.7)	0.072
Microvascular, n (%)	12 (6.6)	4 (13.8)	0.18
Digital ischemia, n (%)	3 (1.7)	4 (13.8)	**<0.001**
Catastrophic APS	8 (4.4)	3 (10.3)	0.18
Lupus anticoagulant, n (%)	149 (82.3)	23 (79.3)	0.70
Triple Positive, n (%)	75 (41.7)	10 (34.5)	0.46

APS, antiphospholipid syndrome; β2GP1, β2 glycoprotein 1; aCL, anti cardiolipin;
